# TReC: Transferred ResNet and CBAM for Detecting Brain Diseases

**DOI:** 10.3389/fninf.2021.781551

**Published:** 2021-12-23

**Authors:** Yuteng Xiao, Hongsheng Yin, Shui-Hua Wang, Yu-Dong Zhang

**Affiliations:** ^1^School of Computing and Mathematical Sciences, University of Leicester, Leicester, United Kingdom; ^2^School of Information and Control Engineering, China University of Mining and Technology, Xuzhou, China

**Keywords:** pathological brain, magnetic resonance imaging, multi-class classification, transfer learning, attention mechanism

## Abstract

Early diagnosis of pathological brains leads to early interventions in brain diseases, which may help control the illness conditions, prolong the life of patients, and even cure them. Therefore, the classification of brain diseases is a challenging but helpful task. However, it is hard to collect brain images, and the superabundance of images is also a great challenge for computing resources. This study proposes a new approach named TReC: Transferred Residual Networks (ResNet)-Convolutional Block Attention Module (CBAM), a specific model for small-scale samples, to detect brain diseases based on MRI. At first, the ResNet model, which is pre-trained on the ImageNet dataset, serves as initialization. Subsequently, a simple attention mechanism named CBAM is introduced and added into every ResNet residual block. At the same time, the fully connected (FC) layers of the ResNet are replaced with new FC layers, which meet the goal of classification. Finally, all the parameters of our model, such as the ResNet, the CBAM, and new FC layers, are retrained. The effectiveness of the proposed model is evaluated on brain magnetic resonance (MR) datasets for multi-class and two-class tasks. Compared with other state-of-the-art models, our model reaches the best performance for two-class and multi-class tasks on brain diseases.

## Introduction

The brain is susceptible to external physical and chemical factors that can lead to damage and death of nerve cells, which can be life-threatening in severe cases (Nayak et al., 2018). Therefore, early diagnosis of pathological brains leads to early interventions in brain diseases, which may help control the illness conditions, prolong the life of patients and even cure them (Lu et al., 2020a). One of the most effective methods in brain diagnosing is neuroimaging, whose modalities consist of MRI, CT, and magnetic resonance spectroscopy (MRS).

An MRI is considered a standard technology in these modalities due to its high quality and widespread availability (Gorriz et al., 2021). However, traditional radiologists only manually judge MR images based on experiences, which is hard to achieve an agreed interpretation due to cognitive differences of radiologists (Lu et al., 2020c). To resolve this, computer-aided diagnosis (CAD), a technology that assists doctors in making diagnoses with the help of computers, has become a research hot spot (Nayak et al., 2020; Senthilvel et al., 2021).

There were many methods related to two-class (binary) classification for MR brain images, aiming to detect them as healthy or pathological. Traditionally, most methods utilized machine learning for the two-class classification of brain images, which mainly extracted the features and then processed them using classifiers. A simple model was presented to classify MR brain images, where the color moments were extracted, and a feedforward network was used as a classifier. The overall accuracy was 91.80% (Nazir et al., 2015). A Wavelet Entropy was proposed to extract the image information, and a Naive Bayes classifier was utilized to detect brain diseases. The result showed that the accuracy was 92.60% (Zhou et al., 2015). A Wavelet Transform was first utilized to extract features, and an optimized FNN *via* the Adaptive Chaotic Particle Swarm Optimization (ACPSO) was used to classify. The accuracy was as high as 98.75% (Zhang et al., 2010). Based on the extractor of the Stationary Wavelet Transform (SWT) and the classifier of a variant of the FNN, an improved model for the two-class task reached an average accuracy of 99.45% (Wang et al., 2015). In contrast, the pathological brain was detected using the extractor of the Ripplet Transform and the classifier of a variant of the support vector machine (SVM), and the model got a high accuracy for the classification (average > 99%) (Das et al., 2013).

In general, many other technologies were available for machine learning to classify brain diseases, and most of them had achieved good performance, while there were still disadvantages. Various methods were used to extract the features of MR brain images manually, which may not work in other datasets. Recently, the convolutional neural network (CNN), an end-to-end intelligence technique that did not require extracted features manually, promised to solve the abovementioned challenges (Xiao et al., 2021). Besides, CNN was used in various traffic, industry, and other fields (Huo et al., 2019; Li et al., 2020; Xiao et al., 2020). In the field of MR brain images classification, scholars made many successful attempts. For example, brain tumors were detected using the deep learning method of CNN (Uthra Devi and Gomathi, 2020). Then, the CNN model got deeper to improve the effectiveness of the classification (Ayadi et al., 2021). Later, the Y-net model was proposed, replacing the traditional convolutional layer with convolutional U-net architecture to detect brain tumors (Hashemzehi et al., 2021). Six datasets were used to train the CNN model and achieved higher performance (Naseer et al., 2021). The multiscale CNN model was proposed to process three spatial image information (Díaz-Pernas et al., 2021).

However, it was challenging to collect a large-scale brain image dataset. Applying the abovementioned CNN methods to MR brain datasets of small-scale samples caused overfitting problems and thus degraded the accuracy. Therefore, scholars researched small-scale datasets and preferred to focus on utilizing transfer learning. Transfer learning was a favorable way of migrating the parameters of a pre-trained model to a new model to aid in training and narrowing the search space for the new model (Panigrahi et al., 2021). For the two-class classification of MR brain images, the Residual Networks (ResNet)-34 was leveraged as a pre-trained model to classify the brain images as healthy or pathological (Talo et al., 2019a). In comparison, the AlexNet was utilized as a pre-trained model to classify (Lu et al., 2019). The MobileNetV2 was utilized, pre-trained on the ImageNet, and three different feedforward methods were introduced as the final layer for classification (Lu et al., 2020c).

For the multi-class classification of MR brain images, fewer methods focused on it. Several existing methods were based on traditional machine learning, and others applied transfer learning to the classification. The Generalized Autoregressive Conditional Heteroscedasticity (GARCH) model was proposed, where the KNN was an extractor and the SVM was a classifier (Kalbkhani et al., 2013). The Fast Curvelet Transform was leveraged to extract features for the classification using an extreme learning machine (Nayak et al., 2018). A model with a deep-stacked sparse autoencoder was proposed to classify five categories of brain diseases (Jia et al., 2019). The AlexNet, Vgg-16, ResNet-18, ResNet-34, and ResNet-50 were utilized as a pre-trained model to classify the brain diseases as normal, cerebrovascular, neoplastic, degenerative, and inflammatory (Talo et al., 2019b).

However, there were still several challenges in the classification of MR brain images. First, traditional machine learning methods lacked stability on different brain datasets. Second, CNN methods may cause overfitting for small-scale brain datasets. Third, limited methods focused on the multi-class classification for brain datasets. Therefore, this study hopes to leverage transfer learning and attention mechanism into the multi-class classification and two-class classification of MR brain images to address these problems. Overall, our main contributions are as follows:

- A Transferred ResNet-Convolutional Block Attention Module (CBAM) model (TReC) is proposed using learning, ResNet model, and attention module, suitable for small-scale brain samples.
- The best layers of TReC are determined through experiments.
- Experimental results demonstrate that the model reaches the best performance for two-class and multi-class tasks on brain diseases.

The remainder of this study is organized into the following sections: section Materials presents the dataset in our experiment, section Methods expresses the models and methods that we proposed, the results are shown in section Results, and section Conclusion offers our conclusion.

## Materials

**Figure 1:**
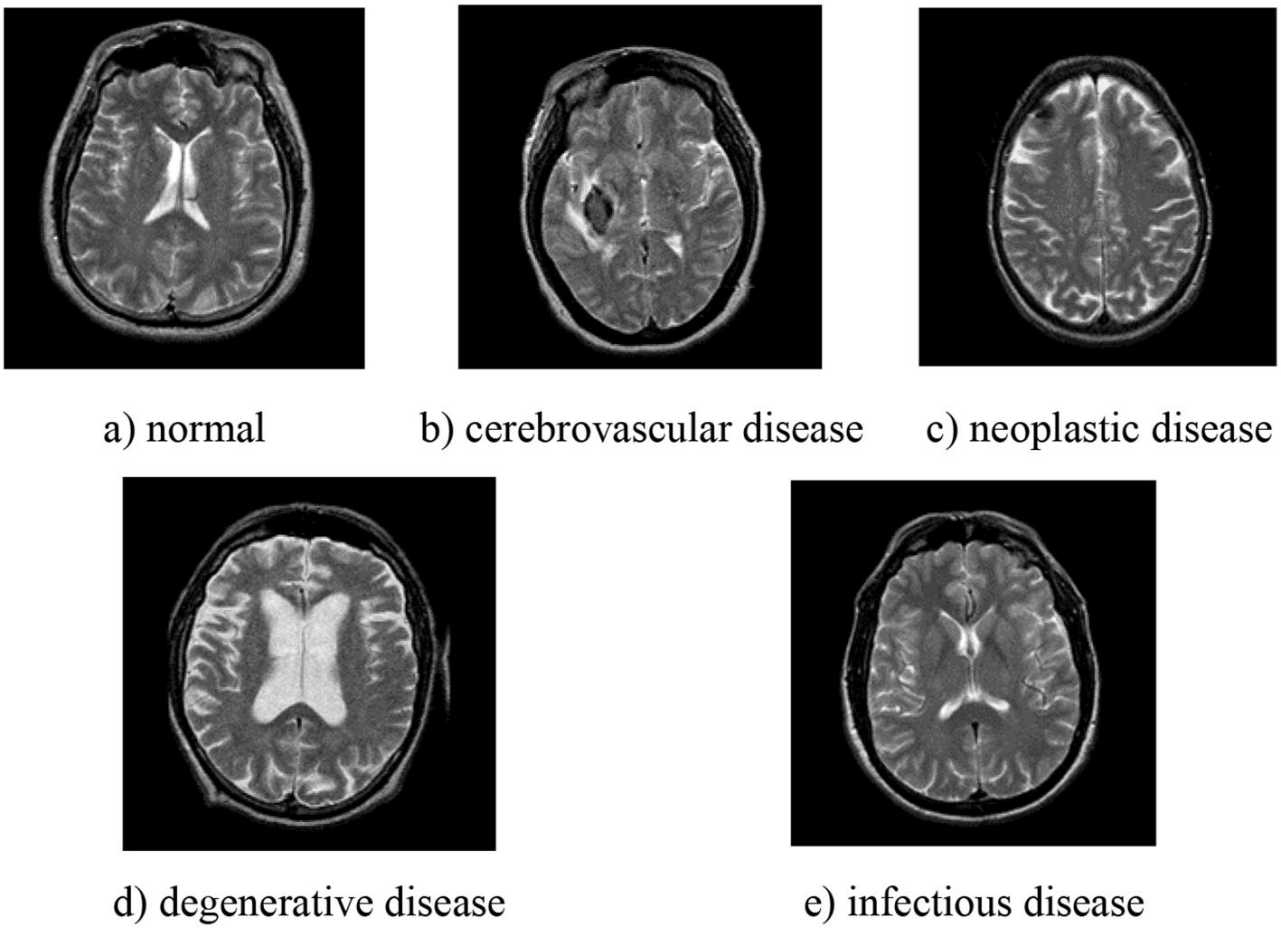
Examples of different types of pathological brain. **(a)** normal; **(b)** cerebrovascular disease; **(c)** neoplastic disease; **(d)** degenerative disease; **(e)** infectious disease.

This study uses the brain MRI dataset from Whole Brain Atlas (http://www.med.harvard.edu/AANLIB/), whose images are in axial orientation and selected by experts. There are five different types in the dataset, namely, normal, cerebrovascular disease, neoplastic disease, degenerative disease, and infectious disease, and some examples are shown in Figure 1. There are 20 images for normal brain, 72 for cerebrovascular disease, 31 for neoplastic disease, 41 for degenerative disease, and 33 for infectious disease. In summary, the normal brain part has 20 images, while the pathological brain parts have 177 images.

## Methods

**Figure 2:**
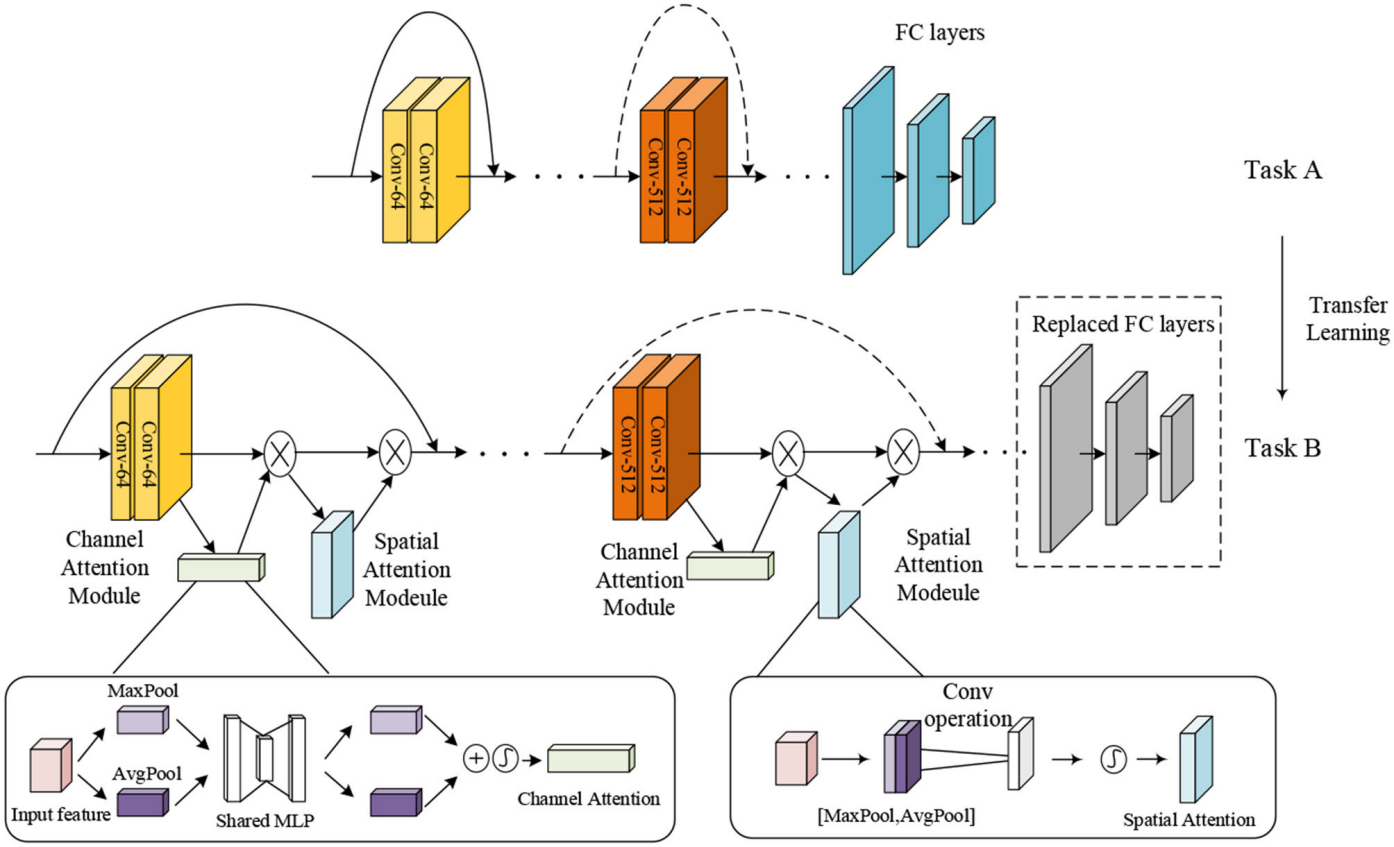
The structure of transfer learning with ResNet-CBAM model.

To improve the performance of multi-class and two-class classification on MR brain images, we utilized TReC based on transfer learning and attention mechanisms to classify brain images. Figure 2 shows the main structure of our model. Specifically, the parameters of the ResNet model, pre-trained on the ImageNet dataset as task A, are served as initialization. Subsequently, a simple attention module named CBAM is introduced, added into every residual block of the ResNet. At the same time, the fully connected (FC) layers of the ResNet are replaced with new FC layers that meet the goal of classification. Finally, all the parameters, such as the ResNet, the CBAM, and new FC layers, are retrained to get more accurate results. It is noteworthy that the parameters of the ResNet are trained based on the initialization. The attention module incorporates the transfer learning model, effectively solving the insufficient number of MR brain images, fully extracting the relevant features of brain images, and avoiding overfitting problems. It can improve the accuracy of the multi-class and the two-class classification for brain images.

### Residual Networks

The ResNet, which introduces residual blocks that are connected across layers, is proposed to avoid gradient disappearance cleverly and speed up learning, arguably one of the most groundbreaking works in the field of computer vision in the past few years (He et al., 2016). Each residual block consists of convolutional layers and a residual connection.

**Figure 3:**
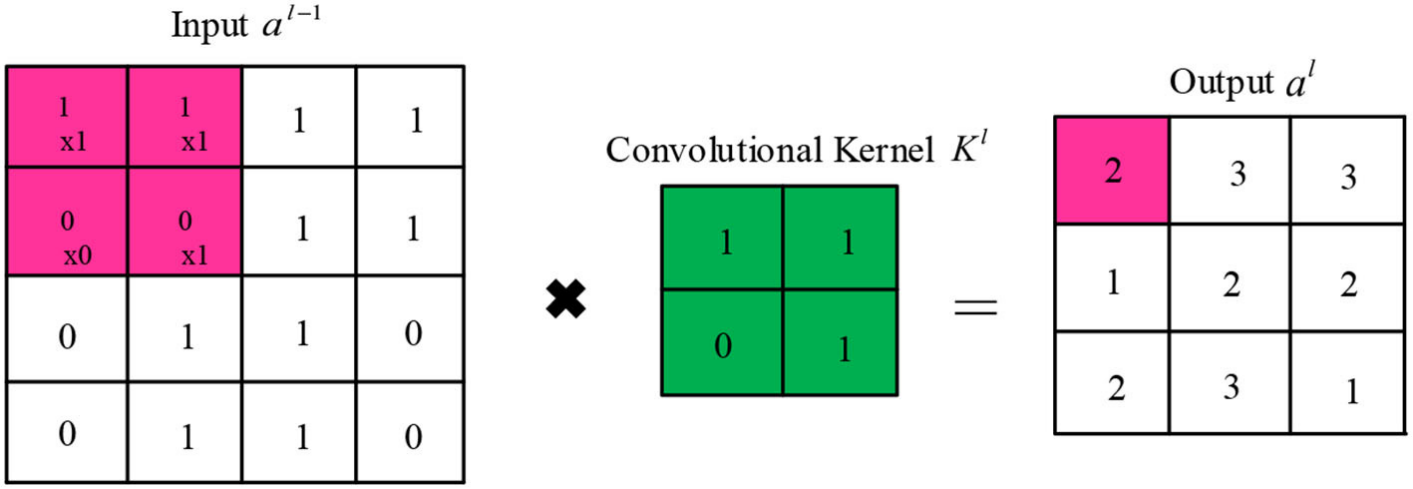
Schematic diagram of the convolutional operation.

In the convolutional layer, each convolutional kernel extracts a specific feature of the input. Several different convolutional kernels are usually used to enhance the richness of the features. The convolutional kernel slides over the feature map in fixed steps and performs dot product operations. Finally, the activation function is added to increase the non-linear expressiveness. The output $a_{j}^{l}$ of the *j*th cell of the convolutional layer*/*is calculated as follows:


(1) $$a_{j}^{l}=f\left(\sum_{i\in M_{j}^{l}}a_{i}^{l-1*}k_{i,j}^{l}+b_{j}^{l}\right)$$

where $M_{j}^{l}$ denotes the selected input feature map set, *k* represents the learnable convolutional kernel, and *f* represents the activation function. As shown in Figure 3, the convolutional kernel *k* can be viewed as a sliding window that slides forward in a set number of steps (stride).

**Figure 4:**
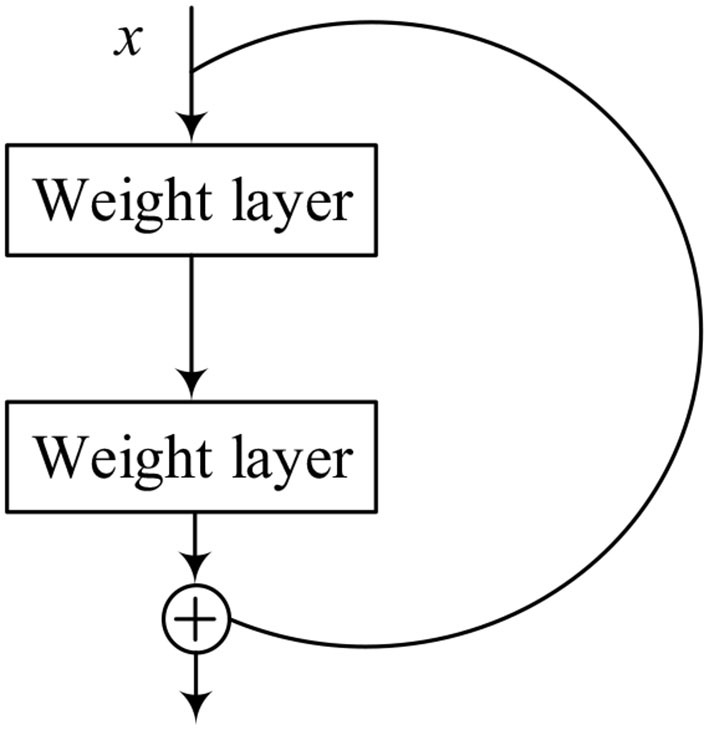
Structure of residual block.

Figure 4 represents the structure of the residual block, which contains a branch that leads to a series of transformations, whose output is added to the input of the block, and it is shown as follows:


(2) H(x)=F(x)+x

where *x* is the input of the structure, and *F* represents the series of the convolutional operations. The ReLU operation is performed before each weight layer in every residual block. ReLU is the activation function, which is shown as follows:


(3) ReLU(x)=max(x,0)

However, an image with a small size may end up after multilayer convolution, which causes the information on the edge of the image to be missed. Therefore, it is necessary to do the padding operation for the input to ensure that all the information is taken into account. The size of padding is related to the size of the convolutional kernel. For a convolutional kernel with edge length, the size of the input image is the same as the output when P=⌊k/2⌋. Only one pooling layer in the ResNet model is usually connected after the last residual block. The primary role of the pooling layer is to filter the features from the convolutional layer to reduce the training parameters and mitigate the overfitting. The output of the pooling layer *l* is expressed as follows:


(4) $$a_{j}^{l}=\text{down}(a_{j}^{l-1}M^{l})$$

where *M*^*l*^ represents the size of the *l*th pooling layer, and down() means downsampling functions, which are Mean-Pooling, Max-Pooling, and Average-Pooling, depending on the aggregation methods. They mean that the most suitable features within the sliding frame are selected as the pooling result while reducing the output size by the factor of *M*. Therefore, the pooling layer drastically reduces the dimensionality of the features, speeding up the training process, and reducing the risk of overfitting.

There are several FC layers of multiple neurons in the last part of the ResNet model. Operations of FC layers are to (i) weight the extracted features and (ii) sum the weighted features. They have three major roles: (1) The integration of the features is learned from convolutional layers and correspondence with label space, (2) the vectorization of the features is extracted by the CNN to transform multi-channel high-dimensional features into one-dimensional vectors, and (3) as a classifier in a classification task, it is possible to integrate all the previous knowledge learned. The FC layers of multiple neurons can approximate any non-linear transformation. At the same time, FC layers play a role in fine-tuning the CNN to enhance the resistance of the model to interference.

### ResNet-CBAM Model

The attention mechanism is derived from the study of human vision, giving the neural network the ability to focus on the subset of the features and select specific inputs. There are two separate submodules in the CBAM, such as the Channel Attention Module (CAM) and the Spatial Attention Module (SAM). As the name implies, the CAM performs channel attention while the SAM takes spatial attention. They save the parameters and computing power and can be integrated into existing networks as a plug-and-play module (Woo et al., 2018).

The details of the CAM and the SAM are shown in Figure 2. The CAM pays more attention to the more critical parts of images, ignoring irrelevant information. At first, the input features are processed in parallel by the average-pooling and the maximum-pooling, which we have introduced in equation (4). Subsequently, the multilayer perceptron (MLP) forwards these two types of data with one hidden layer. Ultimately, the output features are merged by using element-wise summation. In summary, the CAM is expressed as follows:


(5) $$\begin{array}{l} M_{\text{c}}\left(F\right)=\sigma \left(\text{MLP}\left(\text{AvgPool}\left(F\right)\right)+\text{MLP}\left(\text{MaxPool}\left(F\right)\right)\right)\\ \;=\sigma \left(W_{\text{1}}\left(W_{0}\left(F_{\text{avg}}^{\text{c}}\right)\right)+W_{1}\left(W_{0}\left(F_{\max}^{c}\right)\right)\right) \end{array}$$

where **W**_0_ and **W**_1_ are learnable weights, and σ is the sigmoid function, which is expressed as follows:


(6) $$\sigma \left(x\right)=\frac{1}{1-e^{-x}}$$

The SAM is a complement to the CAM, and its primary purpose is to discover where the most meaningful information is after being processed by the CAM. At first, the input features are processed serially by the average-pooling and the maximum-pooling. Then, this information is forwarded by a convolutional layer. The final mathematical representation is expressed as follows:


(7) $$M_{\text{s}}\left(F\right)=\sigma \left(f\left\{\left[\text{AvgPool}\left(F\right);\text{MaxPool}\left(F\right)\right]\right\}\right)$$

where σ represents a sigmoid function, and *f* represents a convolutional operation.

**Figure 5:**
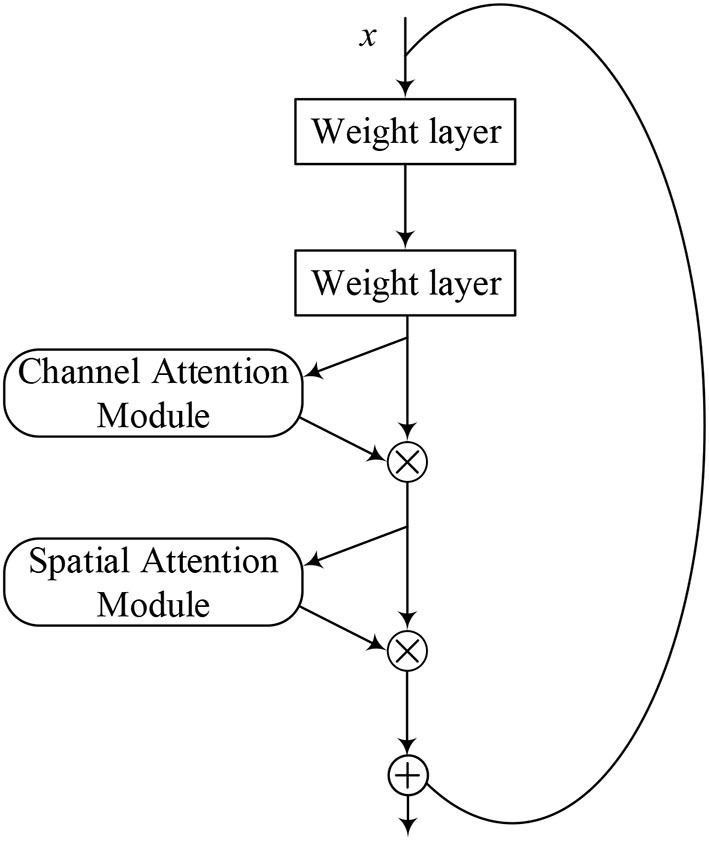
Structure of ResNet-CBAM model.

Figure 5 shows the model structure of the ResNet-CBAM, where the CBAM is merged into a residual block. Same as Figure 4, we used the activation function of ReLU before each weight layer. After extracting the CNN, the layer of the CBAM extracts the most crucial information through the channel and spatial dimensions.

### TReC Algorithm

In this study, we proposed the TReC method. At first, a pre-trained ResNet was employed for MR brain images as initial parameters, and we replaced the last FC layers with new FC layers, which met our task. Then, the parameters of added CBAM layer and new FC layers were trained based on the MR brain images. At the same time, the parameters of the ResNet took the same action based on the initialization, and the algorithm of TReC is summarized in Table 1. We used the cross-entropy function as loss function, which is expressed as follows:


(8) $$\text{Loss}=\frac{1}{\text{N}}\sum_{\text{i }=\;1}^{\text{N}}{\text{L}}_{\text{i}}=-\frac{1}{\text{N}}\sum_{\text{i }=\;1}^{\text{N}}\sum_{\text{c }=\;1}^{\text{M}}{\text{y}}_{\text{ic}}\log({\text{p}}_{\text{ic}})$$

where *p*_*ic*_ shows the predicted probability that the *i*th observation sample belongs to the category *c*. The task is a two-class classification when *M* = 2, and it becomes a multi-class classification when *M* > 2; *M* represents the number of categories to be classified, and *N* represents the number of samples. *y*_*ic*_ is used to check whether *y* equals to *c*. *y*_*ic*_ will take the value of 1, if the true category of the *i*th sample equals *c*. Otherwise, it will take the value of 0.


(9) $$y_{ic}=\{\begin{array}{cc} 1 & \;class\left(i\right)==c\\ 0 & \;\text{otherwise} \end{array}$$

where == is the equality operator.

**Table 1:**
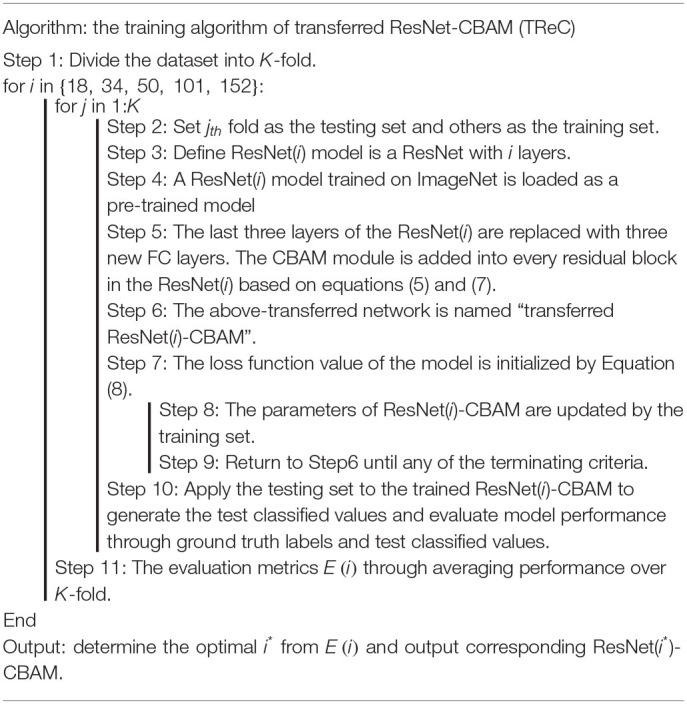
Pseudocode of transferred ResNet-CBAM (TReC) algorithm.

## Results

### Experiment Settings

This study implements the model in Ubuntu operating system, Intel i7-8700K 4* core, 3.7GHz CPU, 64GB memory, and NVIDIA GTX 1080Ti platform. The programming language is Python, and the deep learning framework is PyTorch.

We plan to conduct two experiments on the tasks of multi-class and two-class classifications for pathological brains, respectively. For the two-class classification, the ratio of the pathological brain and normal brain images is very large (20 normal brain images and 177 pathological brain images). Therefore, the normal brain images are copied eight times as 160 images to address the unbalancing data problem. For the multi-class classification, we used the original images without copying.

**Figure 6:**
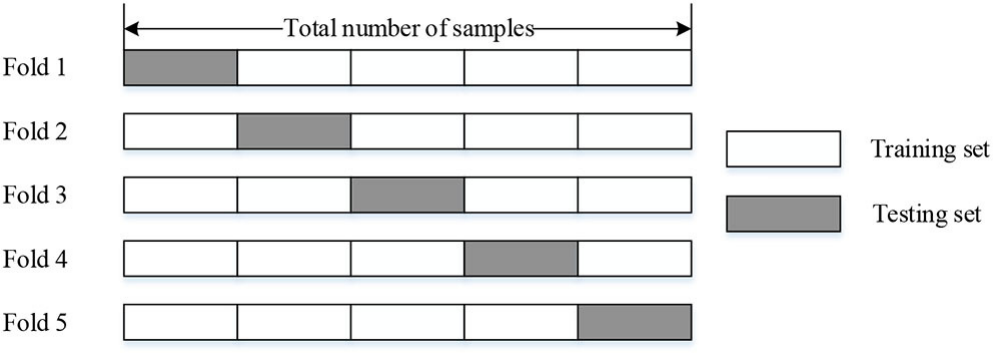
The schematic diagram of 5-fold cross-validation.

We evaluated our model by utilizing 5-fold cross-validation, which means we divided the whole dataset into the training set (counts 80%) and the testing set (counts 20%). The configuration and schematic diagram of 5-fold cross-validation is shown in Table 2 and Figure 6, respectively.

**Table 2:** Configuration of 5-fold cross-validation.

**Task**	**No. of training images**	**No. of testing images**
Two-class	270	67
Five-class	158	39

Finally, we got the final performance by averaging the metrics of 5-fold. We used the metrics of sensitivity, specificity, precision, F1 score, and Overall_accuracy to evaluate the performance of our method. For two-class classification, the equations are shown as follows:


(10) $$\text{Sensitivity}=\frac{TP}{TP+FN}$$

(11) $$\text{Specificity}=\frac{TN}{TN+FP}$$

(12) $$\text{Precision}=\frac{TP}{TP+FP}$$

(13) $$\text{F1-Score}=2\times \frac{\text{Precision}\times \text{Sensitivity}}{\text{Precision}+\text{Sensitivity}}$$

where *TP* is the number of correctly classified pathological brains, and *TN* is the number of correctly classified normal brains. *FP* represents the number of incorrectly classified pathological brains, and *FN* shows the number of incorrectly classified normal brains. Table 3 provides a clearer representation of these four statistics.

**Table 3:** The statistics for two-class classification.

**Classification**	**Ground truth**	
	**Pathological brain**	**Normal brain**
Pathological brain	*TP*	*FN*
Normal brain	*FP*	*TN*

There are five sensitivities, specificities, precisions, and F1-Scores corresponding to five categories for the multi-class classification. We got macro-averaged sensitivity, specificity, precision, and F1-Score based on Equations (10)–(13). The resultant metrics are termed Sensitivity_macro_, Specificity_macro_, Precision_macro_, and F1-Score_macro_, which are expressed as follows:


(14) $${\text{Sensitivity}}_{\text{macro}}=\frac{1}{M}\sum_{c\;=\;1}^{M}{\text{Sensitivity}}_{c}$$

(15) $${\text{Specificity}}_{\text{macro}}=\frac{1}{M}\sum_{c\;=\;1}^{M}{\text{Specificity}}_{c}$$

(16) $${\text{Precision}}_{\text{macro}}=\frac{1}{M}\sum_{c\;=\;1}^{M}{\text{Precision}}_{c}$$

(17) $$\text{F1}-{\text{Score}}_{\text{macro}}=2\times \frac{{\text{Precision}}_{\text{macro}}\times {\text{Sensitivity}}_{\text{macro}}}{{\text{Precision}}_{\text{macro}}{\text{+Sensitivity}}_{\text{macro}}}$$

For simplicity, we utilized the same equation to evaluate overall accuracy for both two-class and multi-class classifications, whose meaning represents the proportion of correctly classified samples to the total samples. It can be expressed as follows:


(18) $$\text{Overall}\_\text{accuracy}=\frac{\mathrm{Total}\text{ number of correct classification}}{\text{Total number of samples}}$$

The training process terminates at 50 epochs, and the batch size is 8. The Adam optimizer is used in each epoch, and the learning rate is 0.001.

### Two-Class Classification for TReC

In this experiment, we compared our method with several state-of-the-art methods such as MobileNet-RVFL-CBA (Lu et al., 2020c), ResNet-ELM-CBA (Lu et al., 2020a), BN-AlexNet-ELM-CBA (Lu et al., 2020b), Deep transfer ResNet (Talo et al., 2019a), and AlexNet+TL (Lu et al., 2019), which all apply transfer leaning into pathological brain detection. The performance is expressed in Table 4. We utilized the ResNet with 18, 34, 50, 101, and 152 layers as pre-trained models, and the results of the evaluation are all the same with 100%. For simplicity, we used TReC to show the three models. It can be found that the accuracies of all methods with transfer learning are not <95%. At the same fold, other evaluation metrics are also relatively high, which proves that the transfer learning method is suitable for small-scale samples, especially for the brain dataset that our method use. Compared with Deep transfer ResNet and AlexNet+TL methods, the results of our method are the same as those of them, reaching the best performance.

**Table 4:** Comparison with state-of-the-art methods for the two-class task.

**References**	**Method**	**Sensitivity**	**Specificity**	**Precision**	**F1-Score**	**Overall_accuracy**
Lu et al. (2020a)	ResNet-ELM-CBA	95.71%	94.29%	–	–	95.00%
Lu et al. (2020c)	MobileNet-RVFL-CBA	98.89%	91.67%	–	–	96.00%
Lu et al. (2020b)	BN-AlexNet-ELM-CBA	97.14%	95.71%	96.17%	96.50%	96.43%
Talo et al. (2019b)	Deep transfer ResNet	–	–	–	–	100.00%
Lu et al. (2019)	AlexNet+TL	100.00%	100.00%	–	–	100.00%
Ours	TReC	100.00%	100.00%	100.00%	100.00%	100.00%

### Multi-Class Classification for TReC

Several transfer learning methods achieve better results for detecting healthy or pathological brain images in the previous section. However, they do not discuss the situation of detecting different categories of brain diseases. In this experiment, we aimed to use our model to classify the multi-class classification of MR brain images.

#### The Determination of Optimal Layers

**Figure 7:**
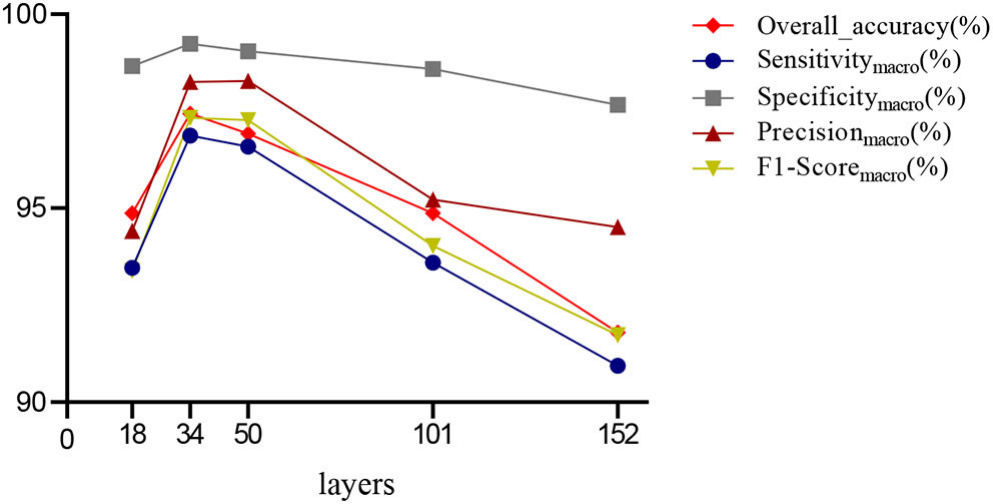
Averaged results for different layers.

First, we used ResNet with 18, 34, 50, 101, and 152 layers as pre-trained models to find out the best model, and the results are shown in Table 5. For clarity, the best performance measures are highlighted with a bold font for each evaluation metric. It can be found that when we utilize the ResNet with 34 layers as a pre-trained model, the Sensitivity_macro_, Specificity_macro_, F1-Score_macro_, and Overall_accuracy all reach the highest value compared with other layers. Although the values for precision are lower than 50 layers, they are close extremely. We can note that the accuracies of 34 layers are the highest for fold-2, fold-3, and fold-5, while for fold-1 and fold-4, the accuracies are very close to the maximum value. Therefore, the best model is the transferred ResNet34-CBAM model, and Figure 7 illustrates these results more clearly. When the convolutional layers are >34, the performance gradually decreases as the number of layers increases, probably because the parameters increase rapidly as the number of layers increases, triggering an overfitting phenomenon for small-scale sample datasets.

**Table 5:** Performance for different folds of proposed model with different layers.

**Model**	**Fold**	**Sensitivity_**macro**_ (%)**	**Specificity_**macro**_ (%)**	**Precision_**macro**_ (%)**	**F1-Score_**macro**_ (%)**	**Overall_accuracy (%)**
Transferred ResNet152-CBAM	1	98.46	99.46	93.33	95.20	97.44
	2	87.56	97.03	94.03	89.53	89.74
	3	79.76	95.20	94.00	84.74	84.62
	4	88.93	96.62	91.19	89.18	87.18
	5	100.00	100.00	100.00	100.00	100.00
	Average	90.94	97.66	94.51	91.73	91.80
Transferred ResNet101-CBAM	1	91.67	97.80	95.33	92.84	92.31
	2	92.14	97.88	93.11	92.32	92.31
	3	97.50	99.33	98.00	97.61	97.44
	4	100.00	100.00	100.00	100.00	100.00
	5	86.67	97.95	89.66	87.39	92.31
	Average	93.60	98.59	95.22	94.03	94.87
Transferred ResNet18-CBAM	1	95.14	98.47	96.03	95.52	94.87
	2	95.14	98.64	96.00	95.41	94.87
	3	96.67	98.86	93.33	94.18	94.87
	4	95.00	99.43	96.00	94.92	97.44
	5	85.33	97.90	90.67	86.91	92.31
	Average	93.46	98.66	94.41	93.39	94.87
Transferred ResNet50-CBAM	1	98.18	99.20	98.67	98.36	97.44
	2	96.67	99.05	98.95	97.64	97.44
	3	97.14	99.20	98.67	97.77	97.44
	4	93.81	98.62	96.36	94.74	94.87
	5	97.14	99.17	98.75	97.82	97.44
	Average	96.59	99.05	**98.28**	97.27	96.92
Transferred ResNet34-CBAM	1	95.00	99.20	98.67	96.45	97.44
	2	100.00	100.00	100.00	100.00	100.00
	3	97.14	99.36	97.78	97.29	97.44
	4	95.56	98.52	95.97	95.36	94.87
	5	96.67	99.13	98.82	97.58	97.44
	Average	**96.87**	**99.24**	98.25	**97.33**	**97.44**

*The bold values indicate the best performance on the particular metrics*.

**Figure 8:**
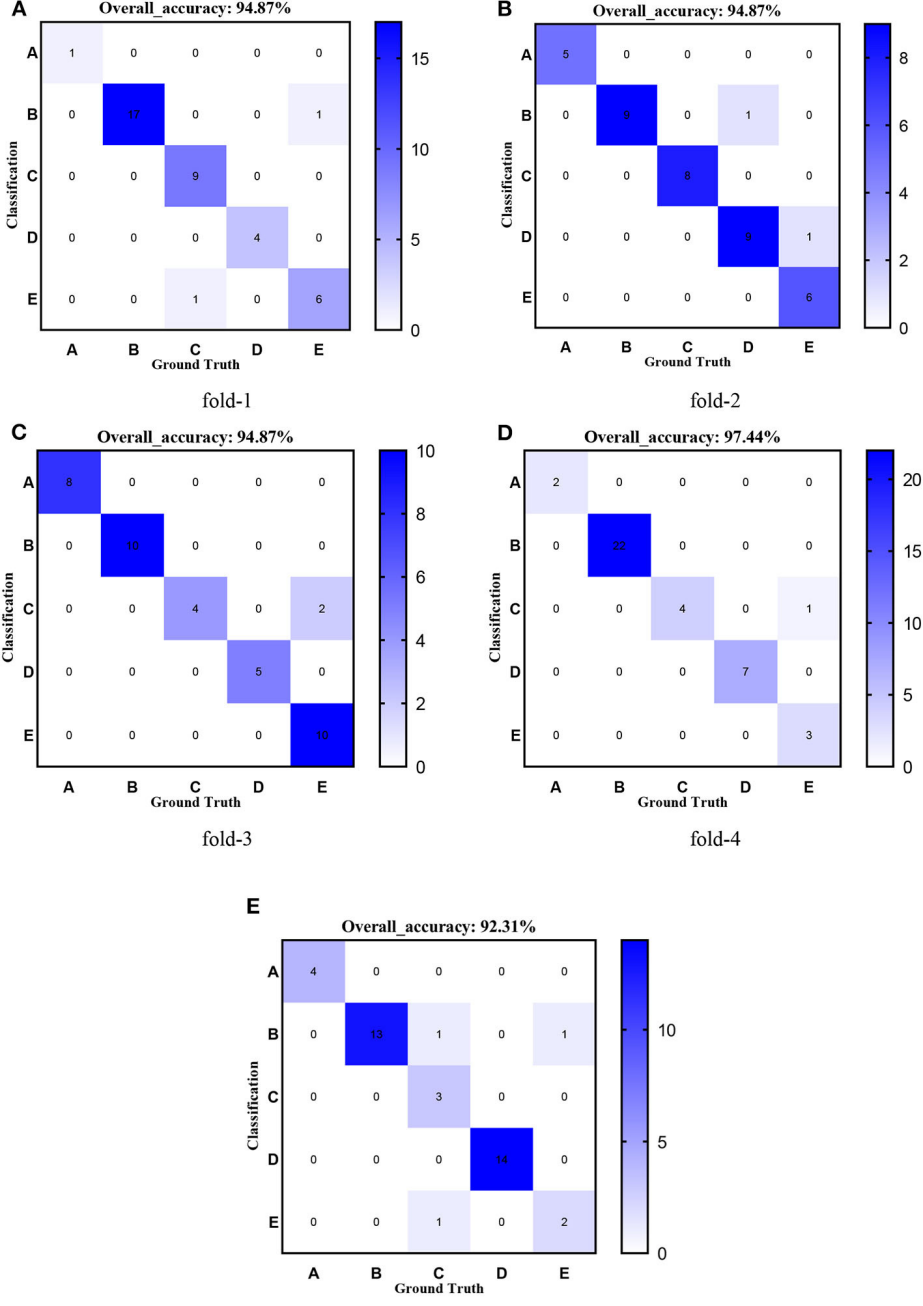
Confusion matrixes of transferred ResNet18-CBAM. **(A)** fold-1; **(B)** fold-2; **(C)** fold-3; **(D)** fold-4; **(E)** fold-5.

**Figure 9:**
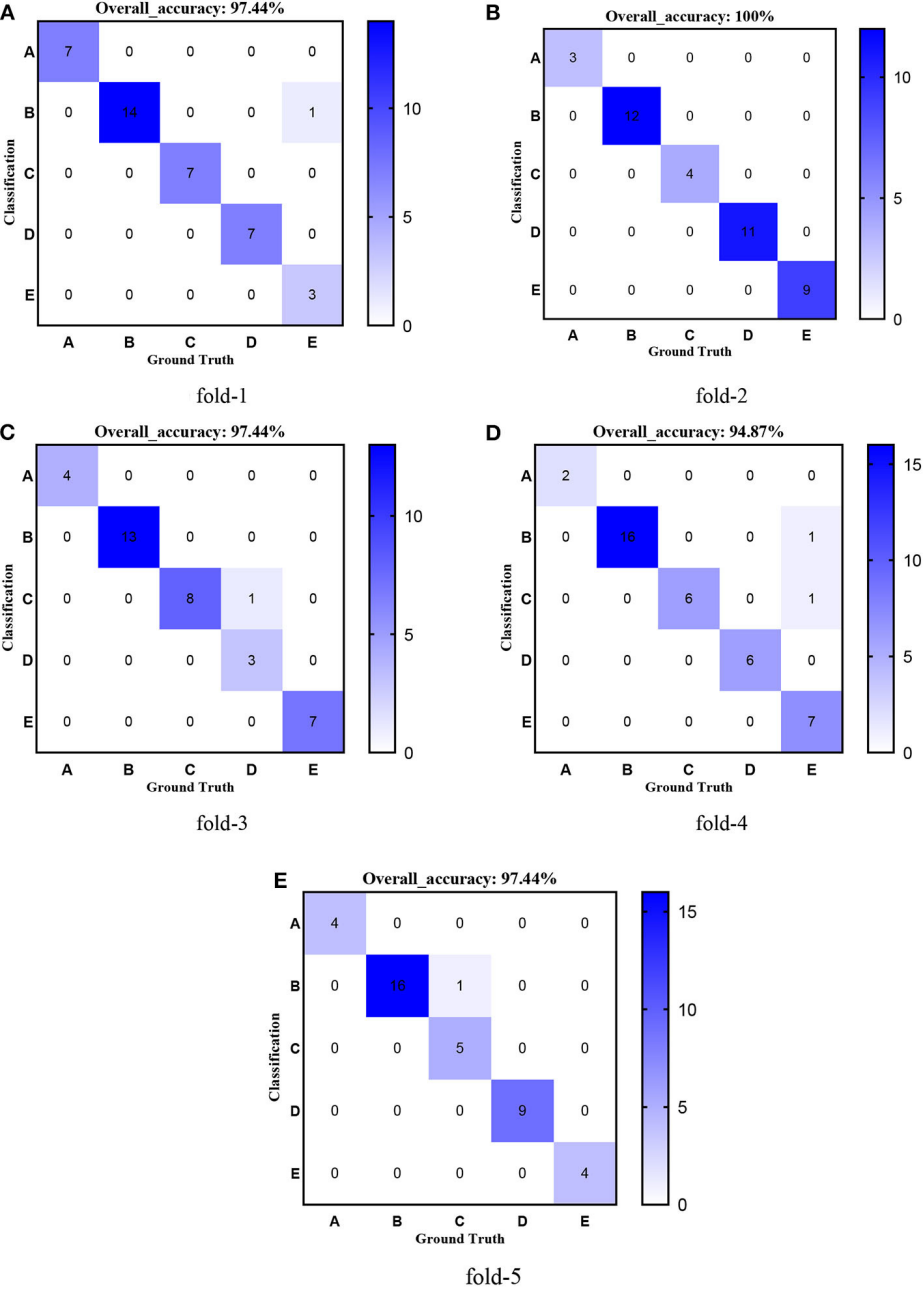
Confusion matrixes of transferred ResNet34-CBAM. **(A)** fold-1; **(B)** fold-2; **(C)** fold-3; **(D)** fold-4; **(E)** fold-5.

**Figure 10:**
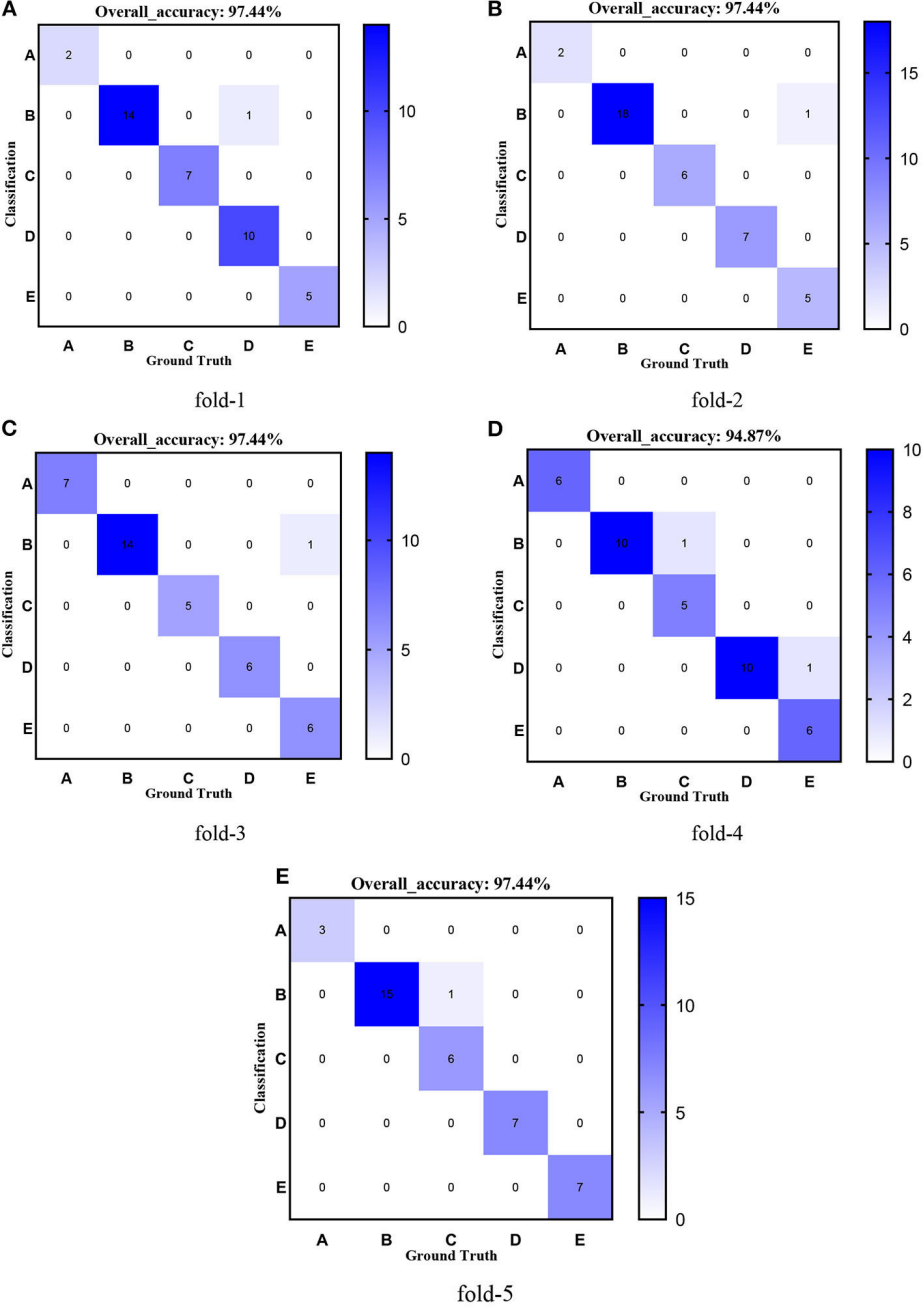
Confusion matrixes of transferred ResNet50-CBAM. **(A)** fold-1; **(B)** fold-2; **(C)** fold-3; **(D)** fold-4; **(E)** fold-5.

**Figure 11:**
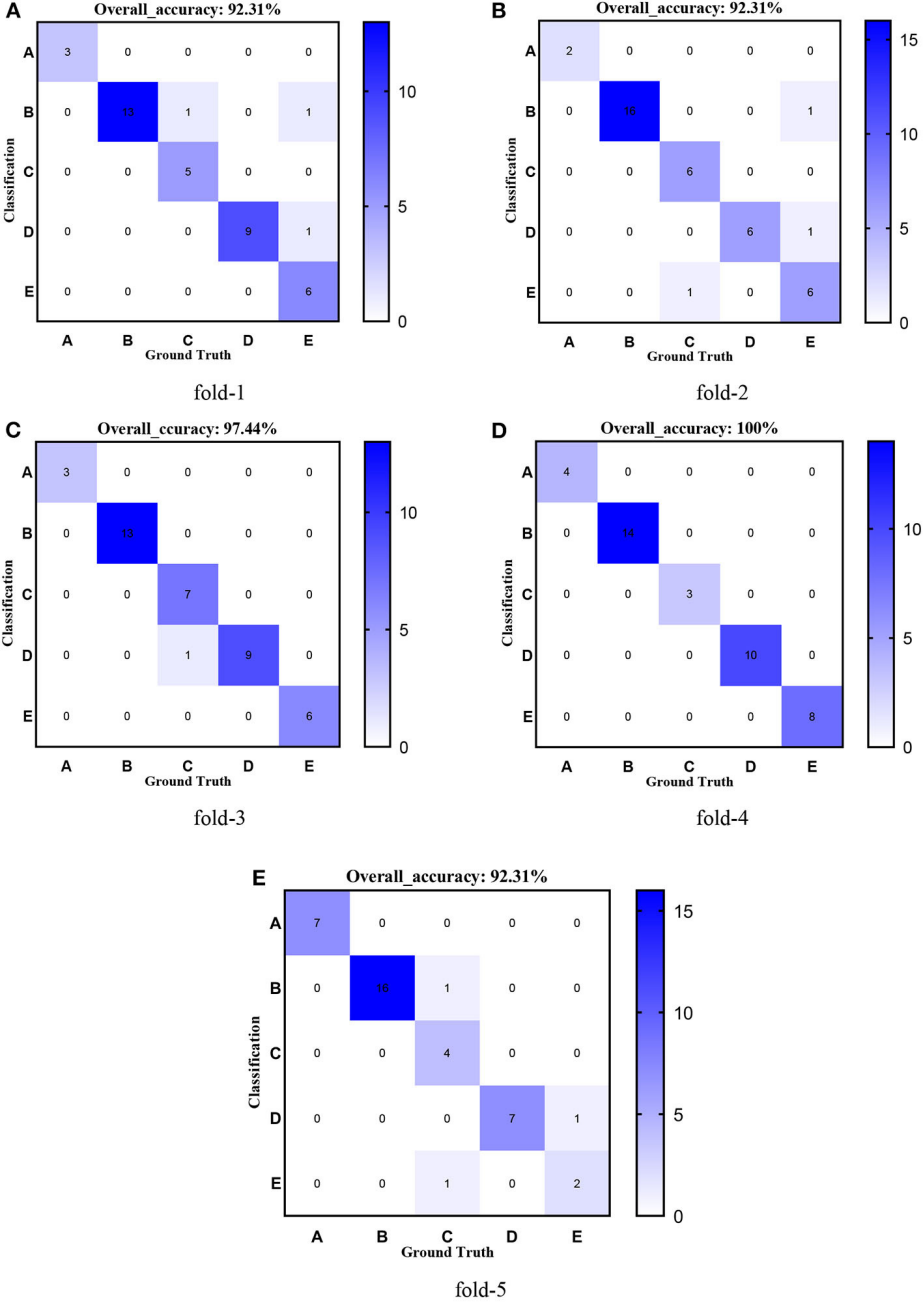
Confusion matrixes of transferred ResNet101-CBAM. **(A)** fold-1; **(B)** fold-2; **(C)** fold-3; **(D)** fold-4; **(E)** fold-5.

**Figure 12:**
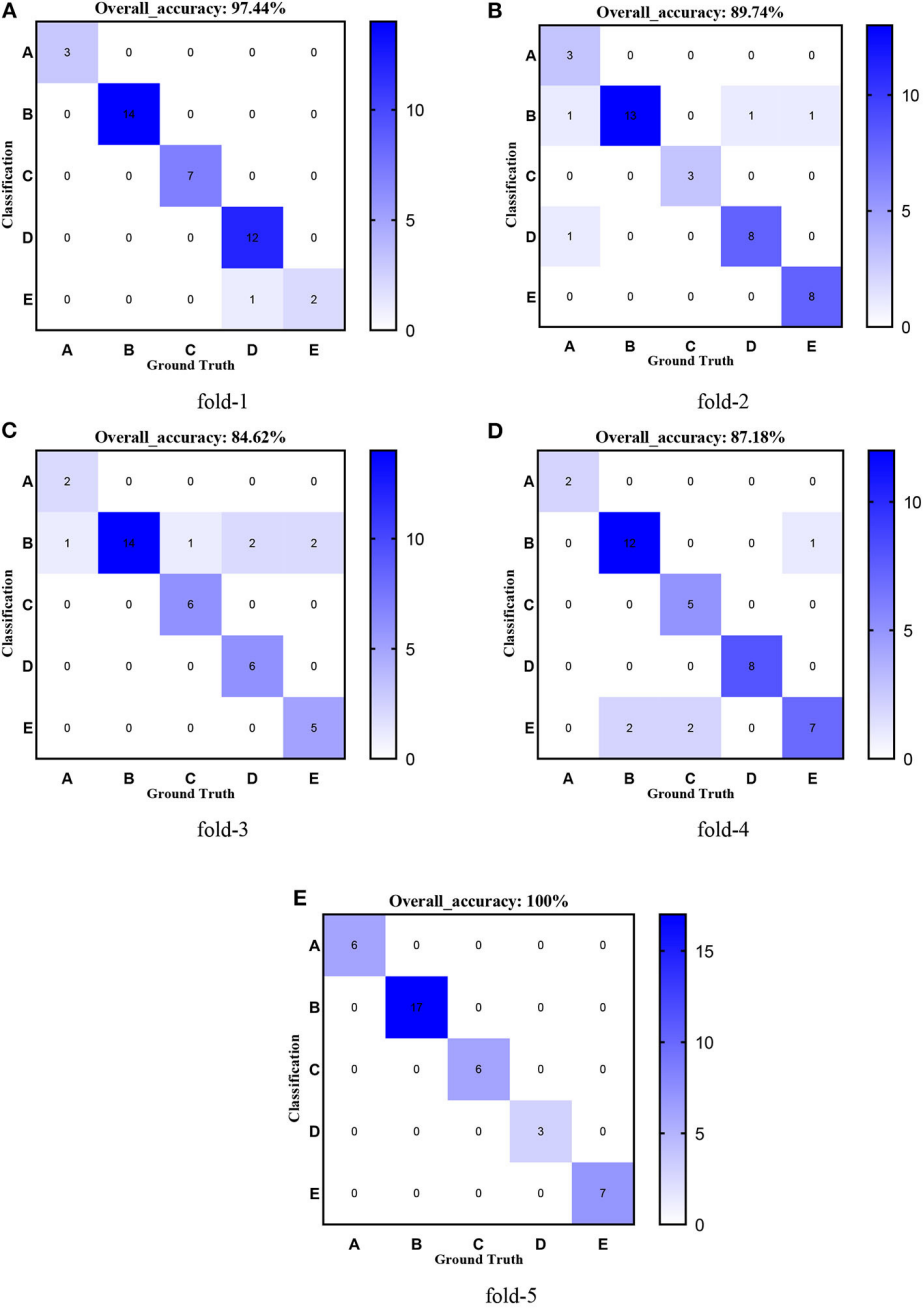
Confusion matrixes of transferred ResNet152-CBAM. **(A)** fold-1; **(B)** fold-2; **(C)** fold-3; **(D)** fold-4; **(E)** fold-5.

Figures 8–12 show the confusion matrixes of TReC with different layers, where A, B, C, D, and E represent normal, cerebrovascular disease, neoplastic disease, degenerative disease, and infectious disease, respectively. The diagonal elements of the matrix represent the number of brain types that the model accurately predicts. They also show that transferred ResNet34-CBAM gets the best performance for multi-class classification. For each fold in transferred ResNet34-CBAM, predictions for the normal and cerebrovascular disease are entirely accurate. At the same time, there are two failed predictions for the other three categories of diseases. The number of mismatched images is the lowest among the five different layer models.

#### Effectiveness of CBAM

**Figure 13:**
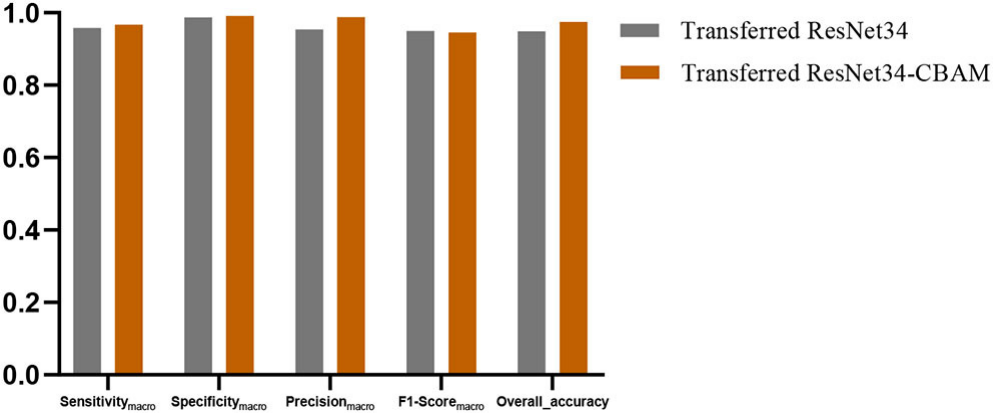
The evaluated metrics between transferred ResNet34 models with and without CBAM.

In this section, we discussed the effectiveness of the CBAM module. Figure 13 shows the averaged Sensitivity_macro_, Specificity_macro_, Precision_macro_, F1-Score_macro_, and Overall_accuracy between transferred ResNet34-CBAM and transferred ResNet34-CBAM. It can be noted that in the five evaluated metrics, all the results for transferred ResNet34-CBAM are better than those for transferred ResNet34, which also implies that the transferred ResNet model with CBAM module classifies all five categories of brain images better than the model without CBAM.

#### Comparison With State-of-the-Art Models

We compared our method with several state-of-the-art methods, where the method of FCEntF-II+K-ELM uses Wrapping-based Fast Curvelet Transform (FCT-WR) as an extractor, kernel extreme learning machine (K-ELM) as a classifier (Nayak et al., 2018), and the study by Talo et al. (2019b) utilized transfer learning with the ResNet50 as a pre-trained model to classify pathological brain. The results are expressed in Table 6. It can be observed that our model is the best of the three methods. In particular, it is noted that our model, better than the transfer learning with the ResNet50, proves the effectiveness of adding the CBAM module.

**Table 6:** Comparison with state-of-the-art methods for the multi-class task.

**References**	**Method**	**Overall_accuracy**
Nayak et al. (2018)	FCEntF-II + K-ELM	93.00%
Talo et al. (2019b)	Transfer learning with ResNet50	95.23%
TReC (Ours)	Transferred ResNet34-CBAM	97.44%

## Conclusion

In this study, we put forward a method of detecting pathological brain using TReC based on transfer learning and attention mechanism. The experimental results show that in the situation of small-scale samples, our model still achieves the state-of-the-art performance for two-class and multi-class classification tasks (accuracy of 100% for the two-class task and 97.44% for the multi-class task).

However, some limitations of this study remain and will be listed in our future work. The interpretation of the proposed model is complex, and the reason for this accurate classification result is unknown. Besides, the dataset we used is small scale. Also, the five brain diseases can be subdivided into more specific diseases, which cannot be classified accurately, such as Alzheimer's disease and motor neuron disease, which belong to the degenerative disease.

We plan to visualize every stage of the model in the future, exploring how the model works. At the same time, we will extend our model to other datasets and improve the performance based on the results. Additionally, we will collect more brain images and further improve our model to detect more specific diseases.

## Data Availability Statement

The original contributions presented in the study are included in the article/supplementary material, further inquiries can be directed to the corresponding author.

## Author Contributions

YX contributed to conceptualization, software, formal analysis, data curation, and writing—original draft. HY contributed to validation, formal analysis, resources, writing—original draft, visualization, and supervision. S-HW contributed to methodology, investigation, writing—review and editing, and funding acquisition. Y-DZ contributed to methodology, validation, investigation, resources, writing—review and editing, supervision, project administration, and funding acquisition. All authors contributed to the manuscript and approved the submitted version.

## Funding

YX holds a China Scholarship (CSC) scholarship with the University of Leicester. This study was partially supported by the Hope Foundation for Cancer Research, UK (RM60G0680), the Royal Society International Exchanges Cost Share Award, UK (RP202G0230), the Medical Research Council Confidence in Concept Award, UK (MC_PC_17171), the British Heart Foundation Accelerator Award, UK (AA/18/3/34220), the Sino-UK Industrial Fund, UK (RP202G0289), and the Global Challenges Research Fund (GCRF), UK (P202PF11).

## Conflict of Interest

The authors declare that the research was conducted in the absence of any commercial or financial relationships that could be construed as a potential conflict of interest.

## Publisher's Note

All claims expressed in this article are solely those of the authors and do not necessarily represent those of their affiliated organizations, or those of the publisher, the editors and the reviewers. Any product that may be evaluated in this article, or claim that may be made by its manufacturer, is not guaranteed or endorsed by the publisher.
